# Weighing the costs: Implementing the SLMTA programme in Zimbabwe using internal versus external facilitators

**DOI:** 10.4102/ajlm.v3i2.248

**Published:** 2014-10-03

**Authors:** Edwin Shumba, Phoebe Nzombe, Absolom Mbinda, Raiva Simbi, Douglas Mangwanya, Peter H. Kilmarx, Elizabeth T. Luman, Sibongile N. Zimuto

**Affiliations:** 1 Zimbabwe National Quality Assurance Programme (ZINQAP) Trust, Zimbabwe; 2 Ministry of Health and Child Welfare (MoHCW), Zimbabwe; 3 US Centers for Disease Control and Prevention (CDC), Zimbabwe; 4 US Centers for Disease Control and Prevention (CDC), United States

## Abstract

**Background:**

In 2010, the Zimbabwe Ministry of Health and Child Welfare (MoHCW) adopted the Strengthening Laboratory Management Toward Accreditation (SLMTA) programme as a tool for laboratory quality systems strengthening.

**Objectives:**

To evaluate the financial costs of SLMTA implementation using two models (external facilitators; and internal local or MoHCW facilitators) from the perspective of the implementing partner and to estimate resources needed to scale up the programme nationally in all 10 provinces.

**Methods:**

The average expenditure per laboratory was calculated based on accounting records; calculations included implementing partner expenses but excluded in-kind contributions and salaries of local facilitators and trainees. We also estimated theoretical financial costs, keeping all contextual variables constant across the two models. Resource needs for future national expansion were estimated based on a two-phase implementation plan, in which 12 laboratories in each of five provinces would implement SLMTA per phase; for the internal facilitator model, 20 facilitators would be trained at the beginning of each phase.

**Results:**

The average expenditure to implement SLMTA in 11 laboratories using external facilitators was approximately US$5800 per laboratory; expenditure in 19 laboratories using internal facilitators was approximately $6000 per laboratory. The theoretical financial cost of implementing a 12-laboratory SLMTA cohort keeping all contextual variables constant would be approximately $58 000 using external facilitators; or $15 000 using internal facilitators, plus $86 000 to train 20 facilitators. The financial cost for subsequent SLMTA cohorts using the previously-trained internal facilitators would be approximately $15 000, yielding a break-even point of 2 cohorts, at $116 000 for either model. Estimated resources required for national implementation in 120 laboratories would therefore be $580 000 using external facilitators ($58 000 per province) and $322 000 using internal facilitators ($86 000 for facilitator training in each of two phases plus $15 000 for SLMTA implementation in each province).

**Conclusion:**

Investing in training of internal facilitators will result in substantial savings over the scale-up of the programme. Our study provides information to assist policy makers to develop strategic plans for investing in laboratory strengthening.

## Introduction

Public health laboratories play a central role in disease detection, prevention and control. High-quality diagnostics and monitoring are critical to ensure better patient outcomes. In response, developing countries have started paying more attention to efforts to expand laboratory capacity, strengthen laboratory systems and improve laboratory quality.^1^ The Strengthening Laboratory Management Toward Accreditation (SLMTA) programme was launched in 2009^2^ and has since been implemented in 47 countries in Africa, Southeast Asia, the Caribbean and Latin America.^3^ The programme has demonstrated measurable improvement in laboratories as a result of an approach that incorporates improvement projects and structured supervisory visits, allowing extension of learning beyond the classroom.^4^

Zimbabwe is a resource-limited country with an annual per capita health expenditure of US$16,^5^ well below the World Health Organization’s recommended minimum of $34.^6^ Inadequate numbers of well-trained laboratory staff and lack of training on laboratory quality management systems are major challenges facing the laboratory system in Zimbabwe.^1,7^ To address these gaps, the US President’s Emergency Plan for AIDS Relief (PEPFAR), through the US Centers for Disease Control and Prevention (CDC), in 2010 made available a five-year grant to the Zimbabwe Ministry of Health and Child Welfare (MoHCW). This funding was earmarked for strengthening laboratory systems through a cooperative agreement with the Zimbabwe National Quality Assurance Program Trust (ZINQAP), a local not-for-profit organisation which focuses on laboratory quality assurance issues in Zimbabwe.

In 2010, the MoHCW’s Department of Laboratory Services embarked on a five-year strategic plan, one objective of which was to implement quality management systems.^8^ The Laboratory Services Directorate adopted SLMTA as a tool for laboratory quality systems strengthening and engaged ZINQAP as the programme provider.

Evaluation of financial costs, or expenditures, of implementing the SLMTA programme is essential in order to guide policy makers and prioritise scarce resources.^9^ Cost analysis is a critical tool in understanding the value of programmes and projecting budgets for scale up.^10^ The aim of this study is to provide a partial financial expenditure analysis of the SLMTA programme in Zimbabwe from the programme provider’s perspective, with a focus on expenditure comparison between using external (international) versus internal (country-based MoHCW) facilitators. These two models have substantial upfront and ongoing cost implications and Zimbabwe has used both in its SLMTA implementation and expansion. We evaluate the financial costs of SLMTA implementation using both external and internal facilitators and project the financial costs of scaling up the programme nationally.

## Research methods and design

### SLMTA programme

Details of SLMTA programme implementation have been described elsewhere.^2^ To date, the SLMTA programme in Zimbabwe has been implemented in three cohorts; only cohorts I (implemented in 2010–2011) and III (implemented in 2012–2013) are included in this analysis. Cohort II was conducted using a different model and detailed cost data were not available. The SLMTA programme in Zimbabwe included four components: (1) baseline audits conducted by ZINQAP staff; (2) three SLMTA workshops of four days each; (3) supervision of the implementation of improvement projects following each workshop; and (4) exit audits. Mentorship, which is often incorporated into SLMTA implementation, was not conducted for these two cohorts during the training because of financial constraints and availability of staff.

Two implementation models were used – one with external workshop facilitators and auditors; and the other with internal workshop facilitators and auditors following extensive training. For cohort I, 11 laboratories were trained using an external facilitator based in Uganda, along with three local facilitators. The exit audit was conducted by three external auditors from Uganda, Lesotho and Botswana. Cohort III used four internal facilitators who were trained in a 13-day training-of-trainers course followed by a six-day auditor training. The exit audit was also conducted by these internal facilitators. This cohort included 19 laboratories (13 in Zimbabwe and six in Namibia).

### Expenditure analysis

A partial expenditure analysis was conducted of the implementation of the SLMTA programme, using both external and internal facilitators. We evaluated the expenditures from a programme perspective, including only direct costs borne by ZINQAP, the programme provider, whilst excluding in-kind contributions and salaries of local facilitators and trainees. Expenditures were collected using a detailed inventory (payment vouchers and general ledger) of all resources associated with the SLMTA programme and the training of local facilitators. Expenditures were tallied for each of the four programme components and then categorised into training equipment, training (facilities and materials), trainers and supervisors (transport; accommodation and per diem; and fees) and participants (transport; accommodation and per diem).

For cohort III, expenditures for supervision and auditing of the six laboratories from Namibia were not available. We therefore estimated costs as though they had been in-country, using an inflation factor based on average expenditures for these activities for the Zimbabwean laboratories. We also calculated the cost of training local facilitators in training-of-trainers and auditing courses. These expenditures were entered into CostIt software version 4.5 (World Health Organization 2007) and analysed in the financial analysis mode. CostIt is software designed to record and analyse economic and financial data. All expenditures are expressed in US dollars, which has been the official national currency of Zimbabwe since 2009.

We used a ‘top-down’ cost accounting approach, which starts with total expenditures and then divides by the number of trained individuals and the number of laboratories to yield the average cost per trained individual and average cost per trained laboratory, respectively.

### Theoretical estimate of expenditures

Expenditures of the two cohorts had considerable inherent variability resulting from contextual factors. For example, the rental cost of the training facility varied from workshop to workshop because of seasonality and fluctuations in the economy. Also, for cohort I, ZINQAP paid per diems to participants, whilst for cohort III, no participant per diem was paid because of budget constraints. To better compare the two models, we estimated SLMTA implementation costs based on a theoretical scenario, keeping all contextual variables constant across the two models. Contextual variables included facility rental fees, training materials, number of laboratories and participants. Expenditure assumptions for this model were based on the expert estimation of ZINQAP programme implementers, using lessons learned from conducting the two previous cohorts and a normative budgeting approach.

### Projected financial costs of national expansion

Outputs for the two models based on theoretical financial costs when holding constant contextual variables were used to project the cost of scaling up the SLMTA programme nationally. We based these estimates on a two-phase implementation plan in which half of the 10 provinces in Zimbabwe would implement SLMTA in each phase. SLMTA would be rolled out in a series of five cohorts (one per province) of 12 laboratories each, for a total of 60 laboratories per phase. For the internal facilitator model, one facilitator training would be held per phase, training four people per province to conduct the SLMTA programme locally. Time required to implement a phase will depend on resources available; we therefore did not specify a timeframe.

## Results

The total expenditures for implementation of the SLMTA programme in 11 laboratories using external facilitators were approximately $64 000 (Table 1). The total expenditures in 19 laboratories using internal facilitators were approximately $28 000, plus $84 000 for facilitator training. This yields an average cost per laboratory of approximately $5800 using external facilitators and $5900 using internal facilitators ($3200 and $4700 per person trained, respectively).

**TABLE 1:** Partial expenditure estimates for the SLMTA program in Zimbabwe.

Item	Cohort 1: External facilitators					Cohort 3: Internal facilitators					Facilitator training	
	Baseline Audits	Workshops	Supervision	Exit Audits	Total	Baseline Audits	Workshops	Supervision	Exit Audits	Total	Facilitator training	Total Internal Plus Facilitator Training
**Training Equipment**	-	-	-	-	-	-	4750	-	-	4750	-	4750
**Training**												
Facilities	-	8380	-	-	8380	-	8093	-	-	8093	15 714	23 807
Materials	-	1630	-	90	1720	-	2040	-	-	2040	6597	8637
**Trainers and Supervisors**												
Transport	293	1300	600	3212	5405	133	200	550	550	1433	2100	3533
Accommodation and per-diem	1218	2300	3800	8342	15 660	-	10 979	855	-	11 834	3455	15 289
Fees	982	-	-	5300	6282	-	-	-	-	-	13 550	13 550
**Participants**												
Transport	-	370	-	-	370	-	-	-	-	-	473	473
Accommodation and per-diem	-	25 992	-	-	25 992	-	-	-	-	-	42 543	42 543

**Total cost $**	**2493**	**39 972**	**4400**	**16 944**	**63 809**	**133**	**26 062**	**1405**	**550**	**28 150**	**84 432**	**112 582**

Number of laboratories trained	-	-	-	-	11	-	-	-	-	19	-	-
Number of participants trained	-	-	-	-	20	-	-	-	-	24	21	-
Average cost of training per laboratory	-	-	-	-	5801	-	-	-	-	1482	-	5925
Average cost of training per participant	-	-	-	-	3190	-	-	-	-	1173	4021	4691

Note: Training Equipment; For the external model, equipment was provided by trainers. >For the internal model, equipment expenses included laptop computer, projector, camera, and printer. Training Facilities; Based on a per-day per-participant rate charged by the facility; 12 days total for the three workshops, 19 days for facilitator training in the internal model. Training Materials; Included binders, stationary, training manuals, etc. Trainers and Supervisors Transport; Baseline audits were conducted by ZINQAP staff using a ZINQAP vehicle provided in-kind; expenditures covered fuel only. For the workshops, expenditures for the external model included flying in trainers; for the internal model, expenditures covered fuel only. For supervision, a ZINQAP veheicle was provided in-kind; expenditures covered fuel to travel to laboratories. Exit audit expenditures included flying in auditors for the external model; for internal model, expenditures covered fuel only. Trainers and Supervisors Accommodation and Per-diem; For cohort 1, ZINQAP staff received accomodation and per-diem when conducting baseline audits. For the external model, hotel expenditures were paid for external trainers for workshops (five nights per workshop), supervision (11 nights), and exit evaluations; per-diems were not paid for workshops, but were paid for supervision and exit audits. For cohort 2, ZINQAP staff were not paid per-diem for conducting baseline audits. For the internal model, hotel expenditures and per-diem were paid for internal trainers as well as three facilitator trainees for the workshops, and were paid for travel to non-local laboratories for supervision and exit audits. For internal model, accomodation and per-diem were paid on a per-person per-night basis for TOT (13 nights) and auditor training (6 nights). Note that hotel rates for Zimbabwe residents are substantially lower than for non-residents. Trainers and Supervisors Fees; For cohort 1, a local consultant was hired to assist with the first baseline audit; remaining baseline audits were conducted by ZINQAP staff at no fee. For the external model, trainer fees were donated for the workshops; fees were $250 per day for exit audits. For the internal model, fees were paid to five trainers for the TOT and two for the auditor training. Participants Transport; For cohort 1, participants were paid transport fees based on mileage to the three workshops; for cohort 2, participant expenses were paid in-kind by their laboratories. For the internal model, participant transport was paid for those not local to the training. Participants Accommodation and Per-diem; For cohort 1, participant accomodation and per-diem were paid at five nights per workshop; for cohort 2, participant expenses were paid in-kind by their laboratories. For the internal model, all participants were required to stay at the training facility; accomodation and per-diem were paid on a per-person basis for TOT (13 nights) and auditor training (6 nights).												

SLMTA, Strengthening Laboratory Management Toward Accreditation

When keeping all contextual variables constant across the two models, the theoretical financial cost of implementing SLMTA in 12 laboratories using external facilitators would be approximately $58 000, whilst the theoretical cost of implementing SLMTA in the same 12 laboratories using internal facilitators would be approximately $15 000, plus $86 000 to train 20 facilitators (Table 2). The estimated financial cost to implement subsequent SLMTA cohorts would remain $58 000 per cohort using external facilitators and would be approximately $15 000 per cohort using the previously-trained internal facilitators, yielding a break-even point of 2 cohorts, at $116 000 for either model.

**TABLE 2:** Theoretical cost estimates for the SLMTA program in Zimbabwe.

Item	External facilitators					Internal facilitators					Facilitator training	
	Baseline Audits	Workshops	Supervision	Exit Audits	Total	Baseline Audits	Workshops	Supervision	Exit Audits	Total	Facilitator training	Total Internal Plus Facilitator Training
**Training Equipment**	-	-	-	-	-	-	-	-	-	-	4200	4200
**Training**												
Facilities	-	10 000	-	-	10 000	-	10 000	-	-	10 000	12 500	22 500
Materials	100	2400	-	100	2 600	100	2400	-	100	2 600	5500	8100
**Trainers and supervisors**												
Transport	500	3000	500	2 550	6 550	500	200	500	500	1 700	2500	4200
Accommodation and per-diem	-	10 020	855	10 020	20 895	-	-	855	-	855	3400	4255
Fees	-	9000	-	9000	18 000	-	-	-	-	-	13 500	13 500
**Participants**												
Transport	-	-	-	-	-	-	-	-	-	-	1000	1000
Accommodation and per-diem	-	-	-	-	-	-	-	-	-	-	43 000	43 000

**Total costs**	**600**	**34 420**	**1355**	**21 670**	**58 045**	**600**	**12 600**	**1355**	**600**	**15 155**	**85 600**	**100 755**

Number of laboratories trained	-	-	-	-	12	-	-	-	-	12	-	12
Number of participants trained	-	-	-	-	24	-	-	-	-	24	20	24
Average cost of training per laboratory	-	-	-	-	4837	-	-	-	-	1263	-	8 396
Average cost of training per participant	-	-	-	-	2419	-	-	-	-	631	4280	4198

Note: Training Equipment: For the external model, assumes equipment will be provided by external facilitators. Includes 1 laptop computer ($1000), 1 projector ($1000), 1 camera ($200), and 1 printer ($2000). Training Facilities: Based on approximately $30 per participant and trainer per day. For workshops, assumes 12 days total for workshops, 28 participants and 4 trainers. For internal model facilitator training, assumes 19 days with 20 participants and 2 trainers. Training Materials: Includes binders, stationary, training manuals, etc. Trainers and Supervisors Transport: For baseline audits and supervision, estimated cost is for fuel to reach the 12 laboratories, assuming a vehicle will be provided by ZINQAP. For the workshops and exit audits, cost for the external model includes flying in trainers; for the internal model, cost includes fuel for trainers to reach the training location and laboratories. For internal model facilitator training, cost covers transport for 5 trainers in the TOT and 2 in the auditing training. Trainers and Supervisors Accommodation and Per-diem: For the workshops and exit audits, cost for the external model is based on $334 per night (US Government rate for Harare, Zimbabwe as of 8/1/2014). Assumes two trainers for five days per workshop (four days per workshop plus one day travel and preparation), and two auditors for 15 days each to conduct exit audits. For internal model, assumes trainers are local to the workshops. For supervision, assumes internal personnel with overnight travel for five of the 20 laboratories, at $175 per night. Note that hotel rates for Zimbabwe residents are substantially lower than for non-residents. For internal model facilitator training, costs were based on expenditures from cohort 3. Trainers and Supervisors Fees: For the workshops and exit audits, cost for the external model is based on $300 per day. Assumes two trainers for five days per workshop, and two auditors for 15 days each to conduct exit audits. For internal model facilitator training, costs were based on expenditures for cohort 3. Participants Transport: For workshops, assumes that trainings will be held in each province; any transport costs will be paid in-kind by the participants’ laboratories. For internal model facilitator training, assumes $25 per participant for each training. Participants Accommodation and Per-diem: For workshops, assumes that trainings will be held locally in each province. For internal model facilitator training, costs were based on expenditures for cohort 3.												

SLMTA, Strengthening Laboratory Management Toward Accreditation.

**FIGURE 1:**
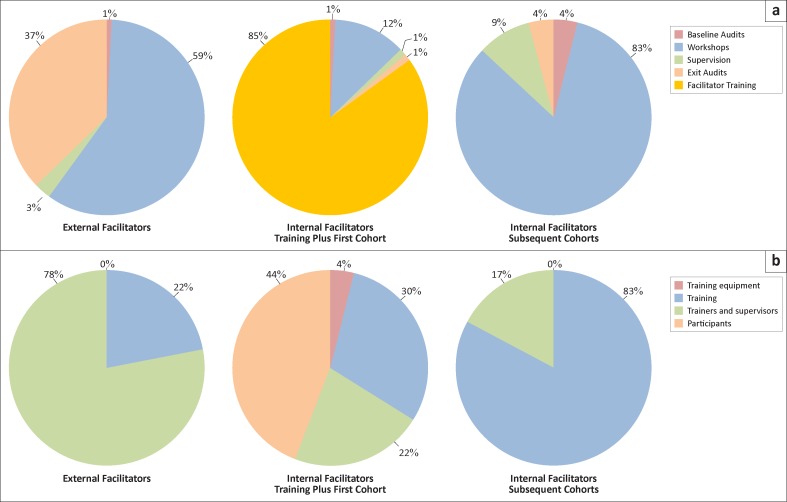
Projected distribution of costs by (a) SLMTA component, and (b) expenditure category.

In the external facilitator model, the majority of the financial costs would be spent on SLMTA workshops (59%) and exit audits (37%) (Figure 1a). For the internal facilitator model, the majority of the cost for the first SLMTA cohort would be spent on conducting the facilitator training (85%), with approximately 12% being spent on conducting workshops. For subsequent cohorts with internal facilitators, the majority of the financial cost would go toward the workshops (83%), with approximately 9% for supervision.

Examining expenditures by expenditure category, the majority of the cost of the external facilitator model would be fees for trainers and supervisors (78%), whilst the remainder would be spent on training (22%) (Figure 1b). For the first cohort using the internal facilitator model, financial costs would be divided fairly evenly between participant expenses (44%), training (30%) and trainers and supervisors (22%). In subsequent cohorts using internal facilitators, training costs would account for the majority of expenditures (83%), with trainers and supervisor expenses accounting for the remaining 17%.

**FIGURE 2:**
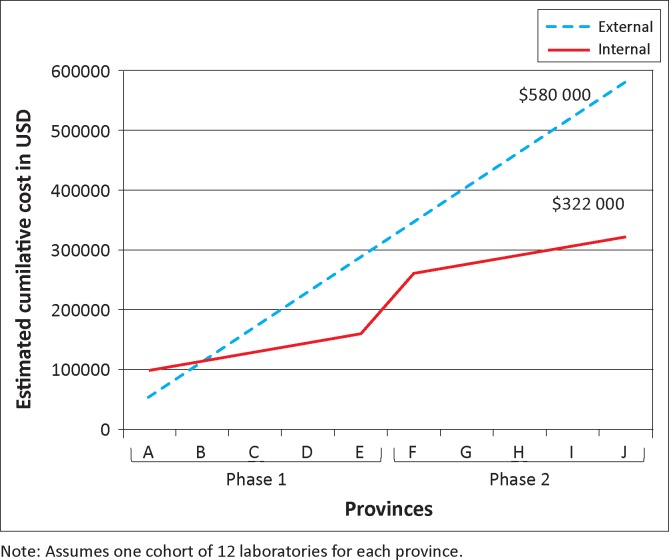
Projected cost of scaling up Strengthening Laboratory Management Toward Accreditation to 10 provinces using external and internal facilitators.

The projected financial cost of scaling up the SLMTA programme nationally is shown in Figure 2. Implementation in 120 laboratories (12 in each of the 10 provinces) would cost approximately $580 000 using the external facilitator model and $322 000 using the internal facilitator model. The initial investment of training 20 internal facilitators ($86 000) will pay for itself by the second SLMTA cohort, after which the programme will benefit from a cost saving of $43 000 (74%) per cohort for the remainder of the cycle.

## Discussion

This is the first detailed expenditure analysis of SLMTA implementation to be published. We found that programme expenditures to implement SLMTA were less than $6000 per laboratory. Yao et al. have shown that SLMTA implementation results in substantial improvements in laboratory quality.^3^ Collectively, Zimbabwe’s cohort I laboratories conduct more than 1.5 million tests each year and their median audit scores more than doubled from pre- to post-SLMTA.^3^ Given the dramatic improvements and relatively low financial cost of SLMTA implementation found in the current study, it can be argued that SLMTA is well worth the investment in time and resources.

Whilst the estimated financial cost to implement a single SLMTA cohort is lower using external facilitators, the upfront investment of training local facilitators pays for itself by the second cohort, resulting in a 44% cost savings over national scale up of the programme. In addition, training local laboratorians to be SLMTA facilitators builds long-term indigenous capacity of the laboratory programme, enabling these individuals to conduct in-country laboratory audits, assist laboratory staff in other activities and improve the overall quality of laboratory management throughout the country. In this case, it is critical to minimise staff turnover between the training and SLMTA implementation so as to ensure a return on investment. Some strategies may include an agreement between the MoHCW and supervisors not to reassign the trainers for a period of 2 years; financial incentives to the participants upon completion of the SLMTA programme; and binding contracts in which the participant agrees to remain on the job for a specified period or until the conclusion of SLMTA implementation in their province. Bringing in external facilitators requires high transport costs as well as facilitators’ fees. Because we assumed that the internal facilitators would be employed by MoHCW and work within their own provinces, these costs would be minimised.

We found that the financial costs of SLMTA implementation are sensitive to participant and trainer travel and per diems. In cohort I, when participants were provided accommodation and per diem, this cost was nearly 40% of the entire financial cost of SLMTA implementation. Hence, decentralising the SLMTA programme to each province, minimising necessary travel for participants, will reduce costs substantially. A study in Cameroon also found that decentralised SLMTA training enables more laboratory staff to be trained, increasing local capacity and sustainability.^11^ Use of internal facilitators will make decentralisation more feasible, as facilitators can be selected strategically from a wide geographic distribution.

SLMTA is often coupled with on-site mentorship in a variety of models so as to boost improvements even further.^12^ Our analysis did not include mentorship costs, because mentorship was not incorporated in the SLMTA programme for these two cohorts. Bringing in external mentors would increase the cost of the programme substantially; however, given that internal facilitators can receive mentorship training, they could also be used in this capacity to reduce costs and have a positive impact on programme implementation.

### Limitations

This study is subject to some limitations. Firstly, several expenses associated with SLMTA implementation, such as overhead costs, vehicles and salaries, were provided in kind and thus not included in this analysis. Our results may therefore underestimate the financial cost of implementing SLMTA in other settings. Also not included was the cost of conducting improvement projects within the laboratories, as this was financed by the individual laboratories; however, SLMTA is designed to focus on management behaviours and participants are encouraged to identify solutions to problems within their existing resources. Expenditures were reported as incurred at the time of implementation, not adjusted for inflation or annuitised over years of useful life. Because inflation has varied widely over the past decade in Zimbabwe,^13^ we did not attempt to adjust for it, but based estimates on approximate costs in 2013–2014. Secondly, there is a level of uncertainty inherent in the assumptions of our theoretical models. However, the resulting financial cost estimates were fairly close to the actual financial costs experienced by the programme, suggesting that they may indeed be realistic. Finally, this analysis is not meant to be an exhaustive assessment of all potential models, but rather a comparison of two models that have been used in Zimbabwe. Additional studies are needed to identify the most cost-effective models for various situations and to conduct an overall economic evaluation of the SLMTA programme. Countries considering the implementation of SLMTA will need to examine the expected costs of each of the various components of programme implementation, taking into account local parameters and potential support from partners.

### Conclusion

Our study suggests that national scale up of the SLMTA programme in Zimbabwe would cost approximately $322 000. Countries considering SLMTA implementation should weigh the pros and cons of investing in training of internal staff to act as programme facilitators. In Zimbabwe, investing in training of local facilitators will result in a nearly 50% decrease in the costs of national expansion, as well as develop in-country capacity of laboratory managers and mentors, supporting programme sustainability.
